# Trends and characteristics of COVID-19 and cardiovascular disease related studies

**DOI:** 10.3389/fphar.2023.1105459

**Published:** 2023-04-25

**Authors:** Ao Cheng, Honghao Ren, Zeyao Ma, Naqash Alam, Linying Jia, Enqi Liu

**Affiliations:** Laboratory Animal Centre, Xi’an Jiaotong University Health Science Centre, Xi’an, Shaanxi, China

**Keywords:** ACE2, omicron, SARS-CoV-2, bibliometrics, cardiovascular disease

## Abstract

**Introduction:** The new coronavirus has caused a pandemic that has infected hundreds of millions of people around the world since its outbreak. But the cardiovascular damage caused by the new coronavirus is unknown. We have analyzed the current global scenario and the general pattern of growth. After summarizing the known relationship between cardiovascular diseases and new coronary pneumonia, relevant articles are analyzed through bibliometrics and visualization.

**Methods:** Following our pre-designed search strategy, we selected publications on COVID-19 and cardiovascular disease in the Web of Science database. In our relevant bibliometric visualization analysis, a total of 7,028 related articles in the WOS core database up to 20th October 2022 were summarized, and the most prolific authors, the most prolific countries, and the journals and institutions that published the most articles were summarized and quantitatively analyzed.

**Results:** SARS-CoV-2 is more infectious than SARS-CoV-1 and has significant involvement in the cardiovascular system in addition to pulmonary manifestations, with a difference of 10.16% (20.26%/10.10%) in the incidence of cardiovascular diseases. The number of cases increases in winter and decreases slightly in summer with temperature changes, but the increase in cases tends to break out of seasonality across the region as mutant strains emerge. The co-occurrence analysis found that with the progress of the epidemic, the research keywords gradually shifted from ACE2 and inflammation to the treatment of myocarditis and complications, indicating that the research on the new crown epidemic has entered the stage of prevention and treatment of complications.

**Conclusion:** When combined with the current global pandemic trend, how to improve prognosis and reduce human body damage could become a research focus. At the same time, timely detection, prevention, and discovery of new mutant strains have also become key tasks in the fight against the epidemic, and full preparations have been made to prevent the spread of the next wave of mutant strains, and still need to continue to pay attention to the differential performance of the variant “omicron.”

## 1 Introduction

Since its discovery in Wuhan, China in 2019, the new coronavirus (SARS-CoV-2) has rapidly spread to the world, infecting billions of people worldwide and constantly affecting human life and economic and technological development. The resulting novel coronavirus pneumonia (Corona Virus Disease 2019; COVID-19), referred to as “new coronary pneumonia,” was named “2019 coronavirus disease” by the World Health Organization. Typical clinical manifestations are fever, chills, cough, chest pain, loss of smell, manifestations of cardiovascular system invasion, acute kidney injury, peripheral nervous system symptoms, etc. (Aleebrahim-Dehkordi et al., 2020; Huang et al., 2020; Ozma et al., 2020). Since the 21st century, there have been three pandemics of infectious diseases caused by coronaviruses: severe acute respiratory syndrome coronavirus pneumonia (SARS) in 2003; Middle East respiratory syndrome coronavirus pneumonia (MERS-CoV) in 2012; Severe Acute Respiratory Syndrome Coronavirus Pneumonia (SARS-CoV). Although humans have had two experiences fighting against the coronavirus pneumonia epidemic, due to the wide spread of the coronavirus itself, the rapid infectivity, and the extremely high pathogenicity, it keeps people from responding immediately. The current coronavirus pneumonia has become one of the most influenza epidemics in human history. Even with the advent of a clinically effective vaccine, there is still no way to effectively control of the epidemic spreading. The emergence of the mutant strain makes the virus spread much faster. It has now entered the third year of the national anti-epidemic, but the specificity of the new coronavirus keeps breaking human perception of the classic coronavirus all the time, which indicates that we must constantly re-examine it from different angles.

The harm of coronavirus to humans has never stopped. The Spike protein (S protein) on the surface of the coronavirus plays a crucial role in the detection and mediation of virus infection by target cell membrane receptors. The S protein of SARS-CoV-2 has become an important target for detection and diagnosis, therapeutic and vaccine development (Hulswit et al., 2016; Li, 2016; Harvey et al., 2021). Both SARS-CoV-1 and SARS-CoV-2 are systemic organs invading viruses whose main organs are in the lungs, and the receptor domain (RBD) of the S protein extends outward and binds with ACE2 which is distributed throughout the human body (mainly in the lungs, and heart) has become a generally recognized pathogenic mechanism, and it is believed to be bound to 449–502aa in the 7KNB structure (Zhou et al., 2020a). According to related articles, “omicron” has a higher affinity with ACE2, which may cause a variant strain to have more serious cardiovascular damage (Li et al., 2022). According to some reported cases, the prevalence rate of cardiovascular disease in COVID-19 patients ranges from 10.2% to 39% (because of the number of cases, incidence of large differences in groups, treatment time, and other underlying diseases), and there is a lot of evidence that the proportion of cardiovascular disease in critically ill patients is significantly higher than that in non-critically ill patients (Argenziano et al., 2020; Huang et al., 2020; Shi et al., 2020; Wu et al., 2020; Zeng et al., 2020). The impact of cardiovascular disease on COVID-19 is unknown; the mechanisms that exacerbate mortality after coronavirus infection need further study; and the impact of neo-coronavirus infection on complications of the cardiovascular system is likewise a focus of research.

Here, we focus on analyzing the current global situation and general pattern of growth of new coronary cases, and after summarizing the known relationship between cardiovascular disease and new coronary pneumonia, we use bibliometric and visualization methods to analyze relevant articles. Summarizing the COVID-19 and cardiovascular linkage provides insights into further research hotspots and directions.

## 2 Materials and methods

### 2.1 Data sources

Data on the number of confirmed cases come from (https://2019ncov.chinacdc.cn/2019-nCoV/global.html) and WHO [WHO Coronavirus (COVID-19) Dashboard | WHO Coronavirus (COVID-19) Dashboard With Vaccination Data], and we counted the confirmed cases that could be collected before 20/10/2022 for analysis. Based on the articles on cardiovascular disease in COVID-19 published in the WOS database (selecting the Web of Science core collection database), the study quantitatively analyzed the corresponding research results using bibliometrics. The online literature search was completed on 20 October 2022.

### 2.2 Search strategies

Articles and reviews were included in the analysis because these document types also included full research ideas and results. In order to ensure that the retrieved articles are comprehensive and accurate, we searched the following keywords: TS = {[(cardiovascular) OR (CVD)] AND [(SARS-CoV-2) OR (COVID-19)]}, and Language = English. All the retrieved publications were filtered to remove duplicate publications. Data entries and collections were verified by two authors (Linying Jia and Ao Cheng).

### 2.3 Data collection

The txt data (the total records of papers from the WOS database, including publication year, title, authors’ names, affiliations, nationalities, abstracts and keywords, journal names, etc.) downloaded from Web of Science was imported into Microsoft Excel 2019 and the VOS viewer. Through several meetings, differences of opinion on the collection were resolved.

### 2.4 Bibliometric analysis

We extracted the number of articles published in each country using Microsoft Excel 2019 (Microsoft, Albuquerque, NM, United States). In addition, contributions from institutions and major journals as well as authors are extracted from the downloaded data and presented in the table. The scientific network also enables the analysis of many publication characteristics, including country and region, institution, time of publication, author and citation frequency.

### 2.5 Visual analysis

**FIGURE 1:**
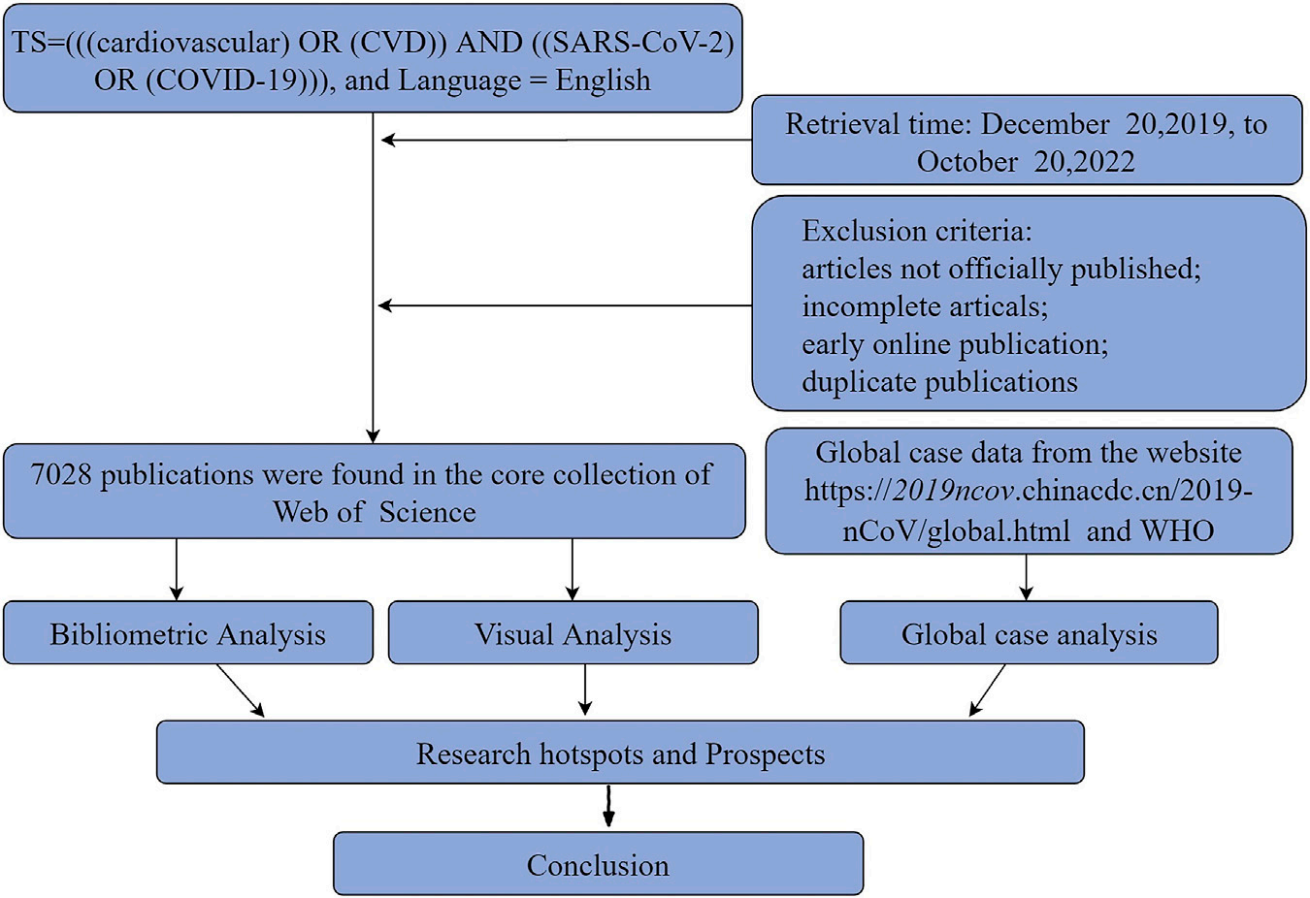
Flowchart of data collection and research procedure.

The data for the analysis of confirmed cases are presented in histograms according to the growth trend, and enumerate the changes in the map for certain countries. The steps of the publication screening and search process are shown in the flowchart (Figure 1).

VOS viewer (Leiden University, Leiden, Netherlands) is a software tool for constructing and visualizing bibliometric networks. These networks include journals, researchers, or individual publications that can form couplings, associations, or partnerships based on citations and bibliographies. The VOS viewer also provides text mining capabilities that can be used to build and visualize collaborative networks of important terms extracted from the scientific literature.

## 3 Results

### 3.1 Comparison of SARS-CoV-1 and SARS-CoV-2

The first outbreak of HCoV was severe acute respiratory syndrome coronavirus (SARS-CoV-1) in 2002 in Foshan, China, and the second occurred in Jeddah, Saudi Arabia in 2016, caused by MERS-CoV Middle East Respiratory Syndrome (MERS-CoV), and the third occurred in Wuhan, China in 2019, Severe Acute Respiratory Syndrome Coronavirus Type 2 (SARS-CoV-2) quickly caused a widespread epidemic worldwide, until today there are still new variants emerging and human infection (Ye et al., 2020). According to existing research, SARS-CoV-1 and SARS-CoV-2 belong to the subfamily Coronaviridae, BetaCoV, and sarbecovirus subspecies in the phylogenetic tree analysis (Kirtipal et al., 2020). From the perspective of viral genetic material, the two genomes are highly homologous, with up to 79% sequence similarity with only 380 base substitutions, and this mutant region may be the difference in their pathogenicity (Petrosillo et al., 2020). The surface of the coronavirus is covered by the spike (s) protein, the membrane (m) protein, and the envelope (e) protein. The RNA in the envelope and the phosphorylated nucleocapsid (n) protein form a helical nucleocapsid, among them, the combination of the spike protein and ACE2 has become the main mechanism of pathogenicity of the two SARS viruses (Wan et al., 2020).

From the point of view of onset, but the incubation period of SARS-CoV-2 (within 14 days, the median is 3–4 days) is longer than that of SARS-CoV-1 (2–10 days, with a median of 4–7 days); the former also has a more extensive mode of transmission than the latter. In addition to the general droplet transmission, there is also aerosol transmission, fecal-oral transmission, and even mother-to-child transmission. This means that under the premise of the same susceptible population, SARS-CoV-2 is more likely to infect a large number of people (Alzamora et al., 2020; van Doremalen et al., 2020). Although the global mortality rate from SARS-CoV-2 (1.05%) is much lower than that from SARS-CoV-1 (10.88%), the huge base of infected people still causes a lot of panic (WHO (2020) Summary Table of SARS cases by country, 1 November 2002–7 August 2003).

The clinical manifestations of SARS-CoV-2 are more diverse than SARS-CoV-1. According to some case reports, some patients with COVID-19 will experience shock, acute myocarditis, chest pain, and even acute myocardial injury (8%–12%), arrhythmia (8.9%–16.7%), heart failure (23%–52%) and other severe acute heart diseases, and the prognosis of these patients is far worse than that of patients without cardiovascular involvement (Bansal, 2020; Zhou et al., 2020b; Diao et al., 2020; Gu et al., 2020; Huang et al., 2020; Kochi et al., 2020; van Doremalen et al., 2020; Author Anonymous, 2021) (Table 1).

**TABLE 1:** Comparison of the characteristics of two coronaviruses.

	SARS-COV-1	SARS-COV-2
Latent period	2–10 days	Within 14 days
Origin	Bats	Bats
Mode of transmission	Close droplets and contact	Close droplets and contact, aerosols in enclosed Spaces and urine, mother-to-child transmission, fecal-oral route
Susceptible population	The population is generally susceptible	The population is generally susceptible
Mortality	10.88% (916/8,422)	1.89% (54,34,118/28,74,41,358)
Clinical feature	Fever	Acute myocardial injury (8%–12%)
	Fatigue	Heart failure (23%–52%)
	Chills	Arrhythmia (8.9%–16.7%)
	Myalgia, cough	Shock
	Nausea	Acute myocarditis
	Diarrhoea	Anorexia, diarrhoea
	Hypocalcemia	Hepatocyte dysfunction
	Lymphocytopenia	Acute kidney injury (mainly severe)
	Acute kidney injury	Peripheral neuropathy
	Peripheral neuropathy	Acute conjunctivitis
		Lymphocytopenia
		Thrombocytopenia
		Coagulopathy

In the reports of five clearly documented complicated cardiovascular diseases in each group, it can be found that the average prevalence of cardiovascular complications in COVID-19 patients is 20.26%, which is significantly higher than that of atypical pneumonia complicated by vascular system diseases (10.10%, Table 2). Then the samples were analyzed by an independent sample T-test, and it was found that (*p* > 0.05, *p* = 0.074), samples from different groups did not show the significance for all the values, which means that samples from different groups showed all the values. It looks like there is no difference, but it cannot be ruled out that the small number of samples affects the interpretation of the results (before averaging and performing T-test analysis, the range data were first taken as medians and replaced with corresponding data).

**TABLE 2:** Manifestations of viral cardiovascular complications.

	Author	Country	Cases	Ratio of CVD
SARS-COV-1	Wang et al	China	76	20.25%
	Li et al.	China	123	9.76%
	Lu et al.	China	801	0.99%–2.62%
	Chan et al.	China	115	9.20%
	Thomas et al.	Singapore	195	4.62%–14.36%
SARS-COV-2	Wu et al.	China	201	19.40%
	Shi et al.	China	416	19.71%
	Huang et al.	China	41	12.63%
	Michael et al.	United States	1,000	10.20%–60.10%
	Zeng et al.	China	416	14.42%

### 3.2 SARS-CoV-2 acts on the cardiovascular system

According to existing research, the current mechanism of SARS-CoV-2 acting on the cardiovascular system can be divided into competitive binding of the virus and ACE2, a cytokine storm, and direct inflammatory damage to the myocardium (Alipoor et al., 2021). Under the induction of multiple effects, patients with underlying cardiovascular disease are more susceptible to infection and become a population at high risk of death (South et al., 2020; Sperotto et al., 2021).

**FIGURE 2:**
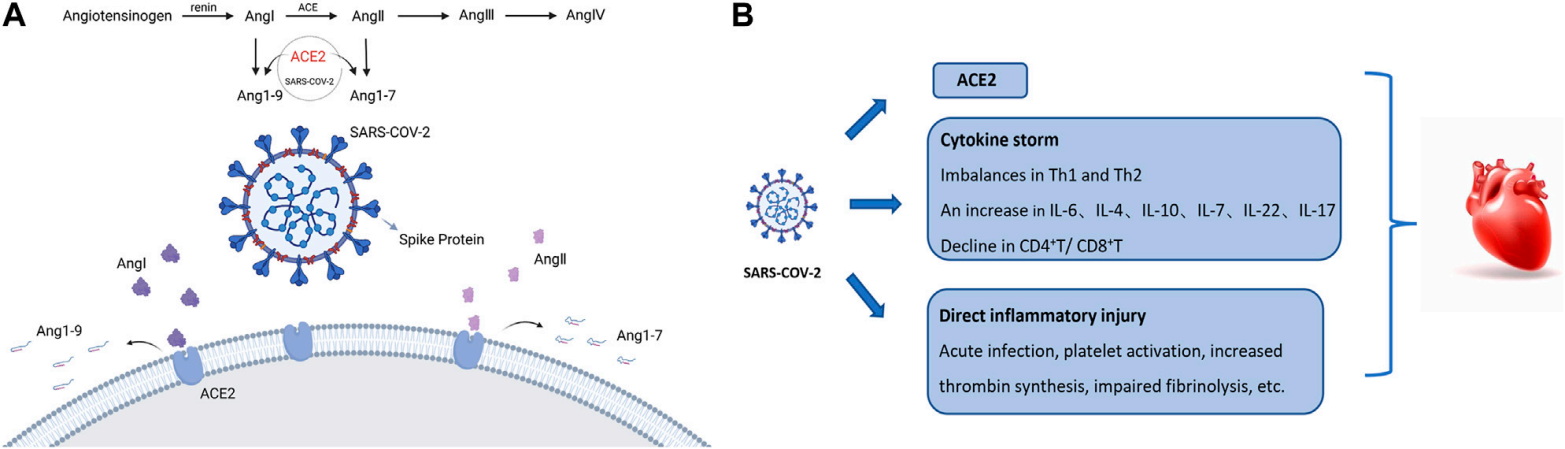
SARS-CoV-2 action on parts of the human body. **(A)** The mechanism of SARS-CoV-2 action with RAS; **(B)** Possible mechanism of SARS-CoV-2 action on the heart. (ACE, Angiotensin converting enzyme; Ang I/II, angiotensin I/II; Ang1-7/Ang1-9, angiotensin1-7/1-9).

The renin-angiotensin system is an important experience regulation system in the human body, and its key substances have a wide range of effects on cardiac muscle, vascular smooth muscle, skeletal muscle, brain, kidney, gonad, and other tissues and organs, which participate in the regulation of target organs, thereby regulating the homeostasis of cardiovascular function, blood pressure, and the maintenance of body fluid and electrolyte balance. When the body reduces renal blood flow or plasma Na+ concentration due to various reasons, angiotensinogen in the liver or tissue is hydrolyzed into Ang I (angiotensin I) by the renin, which is then secreted into the blood by the adjoining glomerular cells. And then ACE (angiotensin-converting enzyme), distributed in the pulmonary vascular endothelium, continues to hydrolyze Ang I into Ang II (angiotensin II), which is an important vasoconstrictor substance in the body. Both Ang I and Ang II can interact with ACE2 to generate Ang1-9 and Ang1-7, which can promote vasodilation, antioxidant effects, and antiproliferative effects, respectively, and produce opposite effects to Ang II (Paul et al., 2006) (Figure 2A).

The spike protein of SARS-CoV-2 binds to human ACE2 (hACE2) on the cell membrane through the S1 subunit of the receptor binding domain (RBD), which is its main pathogenic mechanism, and there are related studies that have shown that the affinity of SARS-CoV-2 RBD to hACE2 is 10–20 times higher than that of SARS-CoV-1 RBD, which may be one of the reasons why SARS-CoV-2 has stronger cardiovascular damage than SARS-CoV-1 (Beyerstedt et al., 2021). The blockade of ACE2 by SARS-CoV-2 leads to the accumulation large amount of Ang II, and the lack of its antagonists, Ang 1-7 and Ang 1-9, leads to an imbalance of the RAAS (renin-angiotensin-aldosterone system), resulting in vasoconstriction, sodium retention, oxidative stress, inflammation, fibrosis, and other disease effects.

In addition, more than 7.5% of cardiomyocytes are ACE2 (+), and the Ang II receptor (angiotensin receptor, AT receptor) is mainly composed of AT1R and AT2R (South et al., 2020). AT1R binds to stimulate the classical effect of Ang II, while AT2R has the effect of antagonizing AT1R (Inciardi et al., 2020). The major distribution of AT1aT in the heart enhanced the effects of angiotensin more pronounced. Some data show that the mutated omicron has a higher affinity for ACE2 than the common type. Although it seems that the clinical manifestations of this type are not serious, the damage to the body is not serious, but it remains to be seen whether there are more serious sequelae. Research, still cannot relax vigilance.

It is generally believed that the imbalance in Th1 and Th2 responses in COVID-19 patients triggers a cellular inflammatory storm, and increase the levels of inflammatory mediators such as interleukin IL-4, IL-10, and IL-6 in tissue samples. Peripheral blood T cell hyperactivation in COVID-19 patients is mediated by increased Th17 and highly cytotoxic CD8^+^ T cells. In addition, the ratio of CD4^+^T to CD8^+^T cells was lower in patients with severe COVID-19 infection than in patients with a mild infection, as well as serum cardiac injury-related proteins, suggesting that inflammatory factors and the cellular inflammatory storm may be one of the causes of heart failure. During the rapid progression of COVID-19, early inflammatory and immune responses also trigger severe cytokine storms.

The inflammatory response first occurs after acute viral infection, before neutralizing antibodies appear. Therefore, this response is thought to be driven by active viral replication, virus-mediated downregulation and shedding of ACE2, and the host’s antiviral response. Secondary inflammatory responses begin with adaptive immunity and neutralization by antibodies. In addition, increased inflammatory activity, platelet activation, increased thromboxane synthesis, and impaired fibrinolysis have been reported to exacerbate myocardial injury after acute infection (Ackermann et al., 2020; Qi et al., 2020; Zhao et al., 2020; Beyerstedt et al., 2021) (Figure 2B).

### 3.3 Global case analysis

**FIGURE 3:**
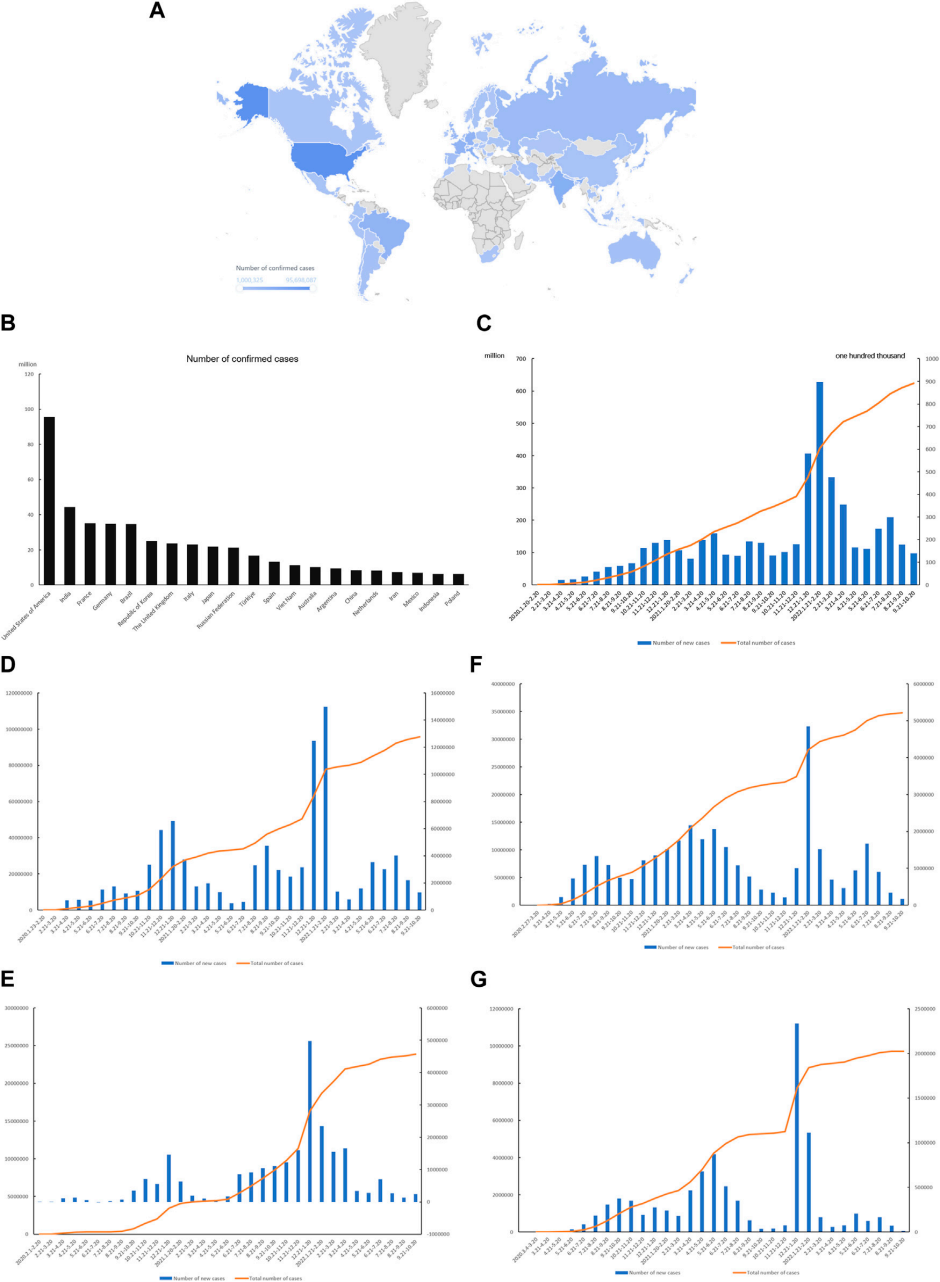
Trends of virus outbreaks in the past 2 years and possible seasonal correlations. **(A)** Map of countries with more than one million cases worldwide (43); **(B)** The top 20 countries in the world for confirmed cases; **(C)** Global case growth trend; **(D)** Increasing trend of cases in U.S.; **(E)** Increasing trend of cases in Britain; **(F)** Increasing trend of cases in Brazil; **(G)** Increasing trend of cases in Argentina.

Until 20 October 2022, there are 62,36,86,859 confirmed cases and 65,55,305 deaths worldwide. There are 68 countries with more than one million confirmed cases, most of which are located in South America, North America, Europe, and Southeast Asia (Figure 3A). Most cases were concentrated in a few countries, with the top 20 confirmed cases accounting for 73.94% of the total number of confirmed cases (Figure 3B). Global cases have continued to increase significantly each month since the outbreak began. It is worth noting that the delta variant (listed as a variant of concern by WHO on 11.5.2021) and the omicron variant (listed as a need for attention by WHO on 26.11.2021), the variants of interest have peak growth after the emergence of each (Figure 3C). By summarizing the monthly number of new cases from 2020 to 2022 in countries with severe epidemics in the northern and southern hemispheres, it can be found that the growth trend of its spread is consistent with the trend of common influenza. With the temperature change, it increases in winter and slightly decreases in summer. However, with the emergence of omicron variant strains, the increase in cases in various places has a trend of breaking through seasonality (Figures 3D–G).

### 3.4 Global publishing trend

**FIGURE 4:**
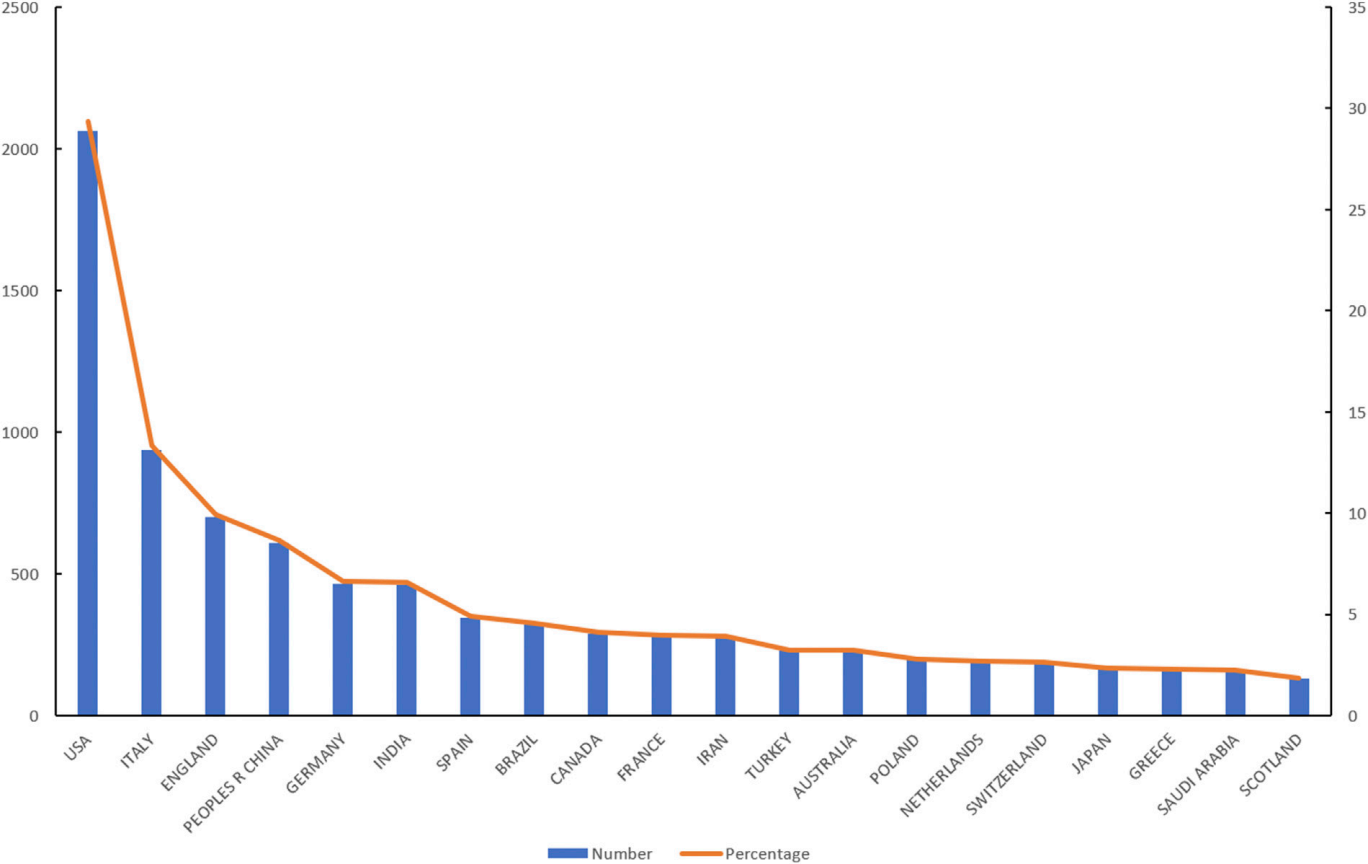
The top 20 countries with the most articles.

Up to 20 October 2022, a total of 7,028 articles and reviews related to cardiovascular disease in English and COVID-19 have been published. The United States published the largest number of articles, a total of 2,064, accounting for 29.37% of the total, followed by Italy (937, 13.33%), England (700, 9.96%), China (610, 8.68%), and Germany (467, 6.65%, Table 3). The 20 countries with the largest number of articles published and their proportions are shown in Figure 4.

**TABLE 3:** The top five countries.

Country	No. (%) of publications (N = 7,028)
United States	2,064 (29.37)
Italy	937 (13.33)
England	700 (9.96)
China	610 (8.68)
Germany	467 (6.65)

Among these articles, the five institutions that published the most articles were Harvard University, University of London, Harvard Medical School, Udice French Research University, and University of California System, and the proportions were as follows: 300 (4.27%), 264 (3.76%), 186 (2.65%), and 173 (2.46%, Table 4).

**TABLE 4:** The top five institutions.

Institution	No. (%) of publications (N = 7,028)
Harvard University	300 (4.27)
University of London	264 (3.76)
Harvard Medical School	186 (2.65)
Udice French Research University	186 (2.65)
University of California System	173 (2.46)

The journals with the most published articles in the world are also cardiovascular Classic journals in the field, such as Frontiers in Cardiovascular Medicine, Journal of Clinical Medicine, European heart journal, International journal of environment research and public health, and Journal of the American College of Cardiology, also ranked among the top five in terms of publication numbers (Table 5).

**TABLE 5:** The top five journal.

Rank	Journal	No. (%) of publications (N = 7,028)
1	Frontiers in Cardiovascular Medicine	131 (1.86)
2	Journal of Clinical Medicine	125 (1.78)
3	European heart journal	99 (1.41)
4	International journal pf environment research and public health	98 (1.39)
5	Journal of the American College of Cardiology	97 (1.38)

Gupta Ankur, Metra Marco, Banerjee Amitava, Mancone Massimo, Banach Maciej, and Chen Juan are the top six authors with the largest number of published related articles, with an average of twenty articles per person (Table 6).

**TABLE 6:** The top six author.

Author	No. (%) of publications (N = 7,028)
Gupta Ankur	27 (0.39)
Metra, Marco	23 (0.32)
Banerjee, Amitava	22 (0.31)
Mancone, Massimo	20 (0.29)
Banach, Maciej	18 (0.26)
Chen, Juan	18 (0.26)

**FIGURE 5:**
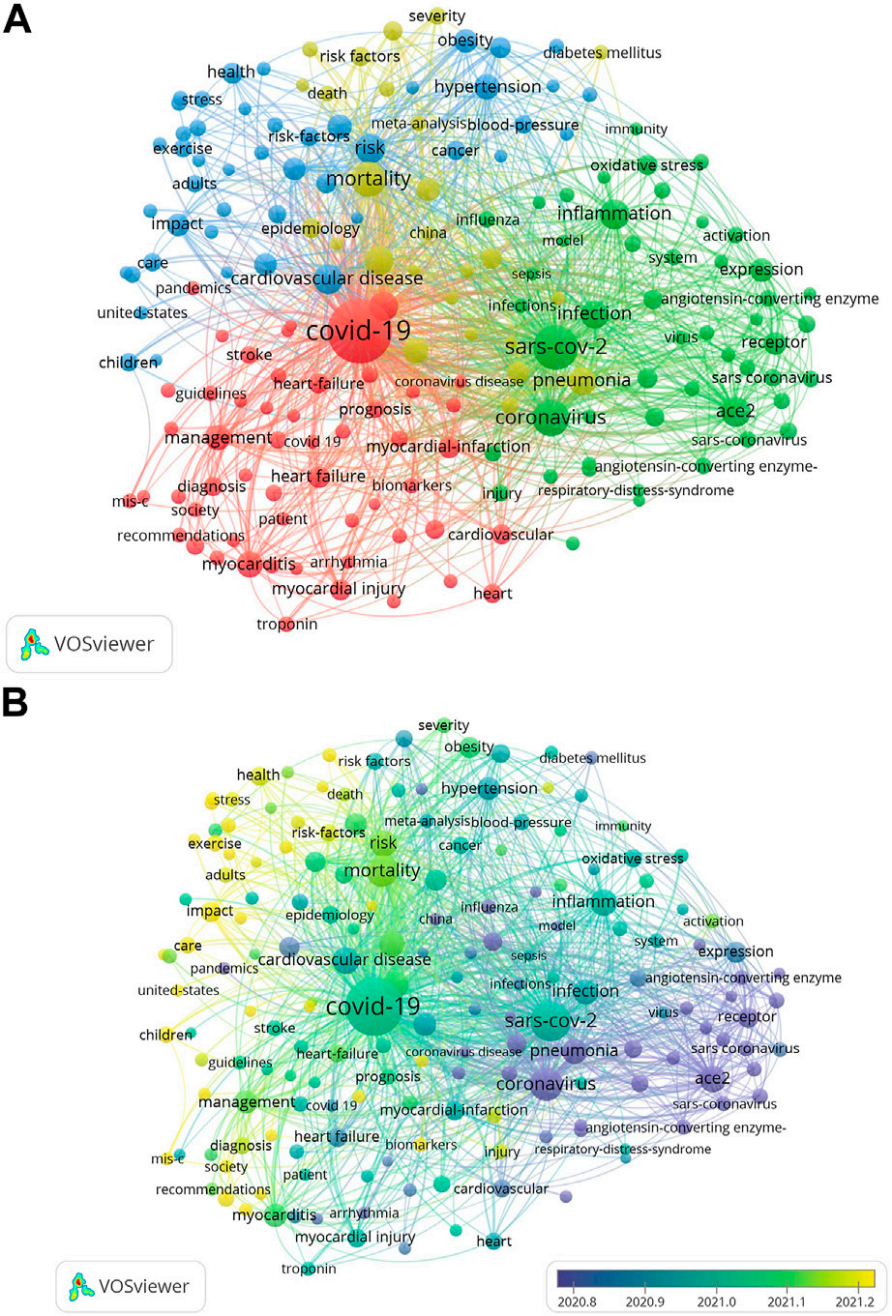
Co‐occurrence analysis of global research about COVID-19. **(A)** Mapping of keywords in the research on atherosclerosis. The size of the points represents the frequency, and the keywords are divided into four clusters: risk factor research (upper in blue), complication research (left in red), cardiac involvement mechanism research (right in green), clinical manifestation research (center in yellow); **(B)** Distribution of keywords according to the mean frequency of appearance. Keywords in purple appeared earlier than those in green, and yellow keywords appeared later. The size of the points represents the citation frequency. A line between two points means that both were cited in one journal.

Co-occurrence analysis refers to the phenomenon of the same type or different types of feature items occurring at the same time. The purpose of co-occurrence analysis is to identify hot areas and directions for collaborative research. This has important implications for the development of scientific monitoring systems and other disciplines. The titles and abstracts of the retrieved articles were opened by the VOS viewer software. The software associates repeated keywords, sets the frequency of the keywords to appear no less than 40 times, and generates an interactive visual analysis image of the selected keywords through the software. We grouped the 176 keywords into four clusters: risk factor research, complication research, cardiac involvement mechanism research, and clinical manifestation research. These results are currently the most prominent subjects of associated cardiovascular disease in COVID-19. In the clustering study of risk factors, the main keywords are cardiovascular underlying diseases such as hypertension. In the Complications study cluster, the main keyword was myocarditis. In the Cluster of Mechanisms of Cardiac Involvement, the main keywords are ACE2 and inflammation. In the clinical performance study cluster, the main keywords were case fatality rate and pneumonia. The size of the VOS viewer keywords in this result is based on the average number of times they appear across all included publications (Figure 5A). In Figure 5B, purple indicates that the keyword appears earlier, and yellow indicates that the keyword appears later. It can be seen that the research direction in the early stages of the disease onset is to explore the mechanisms of SARS-CoV-2 damage to the cardiovascular and cerebrovascular systems. With the clarification of the virus structure and the way it interacts with the human body, the research focus has gradually shifted to the treatment of complications and the clarification of risk factors. In combination with the current global pandemic trend, how to improve prognosis and reduce the damage to the human body may become a research focus.

## 4 Discussion

The new coronary pneumonia epidemic caused by SARS-CoV-2 continues to develop and become more serious around the world. The new coronary pneumonia epidemic caused by SARS-CoV-2 continues to develop and become more serious around the world. While the fatality rate is gradually decreasing, it may also have unknown consequences. Until 20 October 2022, more than 600 million people have been diagnosed worldwide, affecting almost the entire world. The growth trend of the number of cases has a certain correlation with the season or temperature, whether this is due to the general epidemic law of pneumonia or because the occurrence of pneumonia depends on the existence of some of its complications remains to be studied. In particular, the emergence of “omicron” has broken through the original temporal epidemic. In addition to changes in the structure of the virus, there are those possible reasons that may become a major research hotspot. After comparing SARS-CoV-2 with the previous SARS-CoV-1, it can be found that the former is significantly more invasive to the cardiovascular system. However, due to the differences in diagnosis and treatment standards and statistics around the world, we cannot clarify the proportion of the severity of various cardiovascular diseases in COVID-19 patients, thus lacking the support for the specific and reliable case numbers of related mechanisms. The core idea behind the design of the VOS viewer is co-occurrence clustering, which means that if two things appear at the same time, they must be related at some level. Based on the metrics of relationship strength and direction, we can identify current research hotspots and future development trends in this field. We searched the following keywords: TS = {[(cardiovascular) OR (CVD)] AND [(SARS-CoV-2) OR (COVID-19)]}, and Language = English. According to the occurrence network, we have identified four research trends: potential risk factor research, complication research, cardiac involvement mechanism research, and clinical manifestation research. Because of the frequency and time changes of different keywords, the research hotspots have been identified, Complications, and treatment transfer. Since the epidemic is still in the continuous outbreak period, people’s research on its sequelae has not yet opened a chapter, but in the face of higher transmissibility and lower lethal variant strains, whether the patient is truly “healed” after a clinical cure has become an issue hard to avoid. At the same time, timely detection, prevention, and discovery of new mutant strains have also become key tasks in the fight against the epidemic, and full preparations have been made to prevent the spread of the next wave of mutant strains.

## Data Availability

Publicly available datasets were analyzed in this study. This data can be found here: https://covid19.who.int/region/amro/country/.
